# Associations between Medical Student Empathy and Personality: A Multi-Institutional Study

**DOI:** 10.1371/journal.pone.0089254

**Published:** 2014-03-17

**Authors:** Patrício Costa, Raquel Alves, Isabel Neto, Pedro Marvão, Miguel Portela, Manuel João Costa

**Affiliations:** 1 School of Health Sciences, University of Minho, Braga, Portugal; 2 Faculty of Health Sciences, University of Beira Interior, Covilhã, Portugal; 3 Department of Biomedical Sciences and Medicine, University of Algarve, Faro, Portugal; 4 School of Economics and Management, University of Minho, Braga, Portugal; UCLA, United States of America

## Abstract

**Background:**

More empathetic physicians are more likely to achieve higher patient satisfaction, adherence to treatments, and health outcomes. In the context of medical education, it is thus important to understand how personality might condition the empathetic development of medical students. Single institutional evidence shows associations between students' personality and empathy. This multi-institutional study aimed to assess such associations across institutions, looking for personality differences between students with high empathy and low empathy levels.

**Methods:**

Participants were 472 students from three medical schools in Portugal. They completed validated adaptations to Portuguese of self-report measures of the NEO-Five Factor Inventory(NEO-FFI) and the Jefferson Scale of Physician Empathy(JSPE-spv). Students were categorized into two groups: “Bottom” (low empathy, N = 165) and “Top” (high empathy, N = 169) according to their empathy JSPE-spv total score terciles. Correlation analysis, binary logistic regression analysis and ROC curve analysis were conducted.

**Results:**

A regression model with gender, age and university had a predictive power (pseudo R2) for belonging to the top or bottom group of 6.4%. The addition of personality dimensions improved the predictive power to 16.8%. Openness to experience and Agreeableness were important to predict top or bottom empathy scores when gender, age and university were considered.” Based on the considered predictors the model correctly classified 69.3% of all students.

**Conclusions:**

The present multi-institutional cross-sectional study in Portugal revealed across-school associations between the Big5 dimensions Agreeableness and Openness to experience and the empathy of medical students and that personality made a significant contribution to identify the more empathic students. Therefore, medical schools may need to pay attention to the personality of medical students to understand how to enhance the empathy of medical students.

## Introduction

Empathy is a desirable trait in physicians and an important element of the physician-patient relationship [1]. Empathetic physicians have a positive impact on patient satisfaction [2], on confidence in the doctor [3], on adherence to therapy [4], [5] and on clinical outcomes [6], [7]. Empathy is related to understanding patients feelings and, not surprisingly, patients who feel understood are more likely to fully explain their symptoms and to engage in the patient-physician relationship [8]. The multiple definitions of empathy in the medical education literature [9] characterize empathy as a mix of cognitive - understanding patient emotions and communicating the understanding back to the patients - and affective dimensions - emotional responses to patient feelings [10], [11]. The cognitive dimension is amenable to training and therefore an important mission of medical schools is that of caring for and enhancing the empathy of medical students [12]–[15].

The empathy of medical students has been consistently associated with gender and personality [16]–[20]. The Five-Factor Model (FFM or Big5), probably the most accepted personality model worldwide [21], [22], is increasingly being applied in medical education [12], [23], [24]. The FFM postulates five personality dimensions that, altogether, reflect individual differences in social, emotional and behavioral patterns [25], [26]: Neuroticism, Extraversion, Openness to Experience, Agreeableness and Conscientiousness [25]. Conscientiousness includes characteristics such as self-discipline, persistence and striving for achievement. Extraversion consists of attributes like sociability, positive affect and energetic behavior and Agreeableness refers to altruistic affective and collaborative behavior. Neuroticism comprises characteristics like anxiety, fearfulness, and insecurity in relationships. Openness to Experience includes dimensions such as active imagination, preference for variety and intellectual curiosity [27]. A recent multi-institutional study in Australia has shown that student personality profile vary between medical schools [24].

Medical student personality and empathy are associated. The literature reports positive correlations of empathy and sociability [16], Openness to Experience and Agreeableness [18] and negative correlations with Aggression-Hostility [16]. In respect of the Big 5 Model, empathy correlates mostly with Agreeableness [18] probably reflecting this dimension's contribution to interpersonal behavior [28]. Available evidence suggests that high conscientiousness scores in young populations inhibit aggressive behaviors [29], so positive associations should be expected between medical student conscientiousness and empathy.

Most studies that have focused on the connections between student personality and empathy have been restricted to a single institution. Generalization of findings thus requires further multi-institutional design studies. There were two major goals for the present study: (1) the first one was to assess whether associations between medical student's personality dimensions and empathy scores generalize across institutions; (2) the second one was to differentiate students with high empathy scores from the less empathic students.

Thus, we looked for student's empathy scores and personality dimensions from three different schools in Portugal, with different organizations, curricula and admissions processes: i. one school in the south of the country that offers a graduate entry Problem Based Learning (PBL) program that selects students based on a psychological test and Multiple Mini Interviews (MMIs); ii. one school in the center/interior of the country with a horizontally integrated program mostly delivered through tutorials, in groups of 25–30 students that admit most students directly from secondary education, through a national competitive system; iii. one school in the north of the country that offers a systems-based horizontally integrated programs mostly delivered through tutorials with two parallel tracks, a 6 year program for high school entrants and a 4 year program for graduate entry students (annual intake of 18), using a science tests and MMIs.

## Methods

### Ethics

Research in medical education is exempted from the university's Ethical Committee on the ground that this type of research does not have the purpose to answer a research question on health or biomedicine. Nevertheless, this research followed ethical guidelines. Written consent was collected from the participants, prior to the study in accordance with the ethical Declaration of Helsinki. Subjects were specifically informed responses would be kept anonymous, and results would be reported only in aggregate. As all the subjects in the study were adults, there was no need to obtain permission from parents or caretakers. The data collection and the database organization were reviewed and authorized by the Portuguese Commission for Data Protection (CNDP:10432/2011). The study obtained retrospective formal approval from our Ethics review board prior to publication - Subcomissão de ética para as ciências da vida, process SECVS - 071/2013.

### Participants

The study sample comprised 472 first year medical students, from three of the eight medical schools in Portugal, namely from the University of Beira Interior (UBI), 154 (32.6%; response rate = 81.2%), the University of the Algarve (UAlg; response rate = 87.1%), 71 (15%) and the University of Minho (UM), 247 (52.3%; response rate = 87,3%). 370 of the participants (78.4%) were admitted directly from secondary education into 6-year medical degree programs (UBI and UM), whereas 102 (21.6%) were admitted to graduate entry programs (UAlg and UM).

Three entering classes are represented in the study sample, where 312 (66.10%) of students were females. Mean age of 21 years old. A sub- sample of 334 students was selected to compare the students with the highest (Top tercile, *M* = 121.9; *SD* = 8.6) and the lowest (Bottom tercile, *M* = 97.8; *SD* = 5.6) empathy scores (Table 1). These two groups differ significantly in the JSPE-spv scores [*t _(280.3)_* = 30.4, p<.001].

**Table 1:** Study population by gender, university and empathy scores.

	Top tercile (N = 169)	Bottom tercile (N = 165)	Total (N = 334)
	Frequency (%)	Frequency (%)	Frequency (%)
Gender			
Females	120 (71)	94 (57)	214 (64)
Males	49 (29)	71 (43)	120 (36)
Age	21.6 (5.2)	20.7 (4.9)	21.2 (5.1)
University			
UBI	45 (27)	70 (42)	115 (34)
UALG	34 (20)	17 (10)	51 (15)
UM	90 (53)	78 (47)	168 (50)
JSPE-spv	121.9 (5.6)	97.7 (8.6)	110.0 (14.1)

### Instruments

The five personality dimensions, Neuroticism, Extraversion, Agreeableness, Openness to Experience and Conscientiousness, were measured with the Portuguese version of NEO-FFI inventory [30]. It uses a 5-point *Likert* scale ranging from 0 (strongly disagree) to 4 (strongly agree) and can be completed in approximately 15 minutes. The Portuguese version of the NEO-FFI includes 60 items similar to the original North American instrument and corroborates the well- established cross-cultural reliability, factorial structure and the communalities of personality according to gender, age and educational differences [30].

Empathy was measured with the self-administered Jefferson Scale of Physician Empathy (JSPE) – students Portuguese version (JSPE-spv) that includes 20 items answered on a *Likert* type scale: from 1 (Strongly disagree) to 7 (strongly agree), and aggregated in 3 factors: “Perspective Taking” (10 items), “Compassionate Care” (8 items) and “Standing in the Patient's Shoes” (2 items). The JSPE-spv has valid psychometric properties [31].

## Procedures and Data Analysis

In each institution, students were invited to take part in the research by one of the researchers in person. In two institutions students answered at the end of scheduled class time, with the authorization of faculty. In the other institution, students filled the instruments at the end of a welcoming session by the Medical Education Unit. There was no set time limit to answer the forms in any of the institutions. Participation was voluntary and individual and students were ensured they would not be penalized for not participating The researchers guaranteed data would be kept confidential. Written informed consent was obtained from all participants. Students answered the instruments on paper in two schools and online in a computer lab in the other school. Answers were collected during the initial weeks at medical school, so it is highly unlikely that their personality and empathy scores have been influenced by medical school. Data were analyzed with software *STATA 12*.

Empathy was analyzed as a scale variable (continuous variable) for the correlation analysis between the big five personality dimensions and empathy scores and as a categorical variable for the logit regression analysis. Students were categorized into two groups: “Bottom” (low empathy, N = 165) and “Top” (high empathy, N = 169) according to their empathy JSPE-spv total score (the top and the bottom terciles in terms of JSPE-spv scores). The categorization into these two groups was made considering that the second goal of this study was to differentiate medical students on their empathy JSPE-spv scores. Therefore, the students at the extremes could be more easily differentiated on their personality dimensions than those with intermediate self-reported empathy. In order to explore the predictive power of personality to student's empathy we conducted a logit regression analysis on two panels of variables: in panel A we included gender, age and university as predictors of students' empathy and in the panel B the big five personality dimensions were added to the previous predictor variables. The outcome variable assumed the value 1 if the student belonged to the Top empathy group and the value 0 otherwise. Besides regression coefficients, odds ratio and measures of model fit (Nagelkerke pseudo-R^2^, AIC, BIC) we also calculated measures of classification (hit rate, specificity, sensibility, improvement over chance index, ROC curves and optimal cut-off value). A comparison between Panel A and Panel B models was conducted using the logit regression models and the ROC curves.

The distribution was not normal, as a significant Kolmogorov-Smirnov test was found for all continuous variables. Nevertheless, skewness and kurtosis analysis showed no severe departures from normal distribution. Except for age, all skewness and kurtosis absolute values were below 2.

## Results

### Descriptive and Correlation Analysis

For a total of 334 students, we found significant and positive correlations between total JSPE-spv score and Extraversion (r = .183, p<.001), Openness to Experience (r = .216, p<.001), Agreeableness (r = .310, p<.001) and Conscientiousness (r = .188, p<.001). The magnitudes of correlations between personality dimensions and scores of self-reported empathy were low, ranging from −.002 to .310 for Neuroticism and Agreeableness respectively (Table 2).

**Table 2:** Descriptive and Correlation Analysis.

	Neuroticism	Extraversion	Openness	Agreeableness	Conscientiousness
Total Score in the JSPE-spv scale	−.002	.183***	.216***	.310***	.188***
Neuroticism		−.372***	−.194***	−.247***	−.286***
Extraversion			.215***	.400***	.261***
Openness				.144**	−.310***
Agreeableness					.379***
Total Mean (SD)	21.1 (7.7)	31.7 (5.9)	29.7 (5.5)	34.7 (5.3)	35.1 (6.3)
Bottom Group - Mean (SD)a)	21.5 (7.5)	30.7 (6.1)	28.3 (4.5)	33.2 (5.4)	33.7 (6.5)
Top Group - Mean (SD)a)	20.7 (7.8)	32.7 (5.9)	31.1 (6.1)	36.1 (4.7)	36.5 (5.9)
UBI-Mean (SD)b)	20.8 (7.3)	31.9 (6.3)	28.7 (5.9)	34.9 (5.8)	34.5 (6.3)
UALG-Mean (SD)b)	18.7 (6.4)	32.2 (6.0)	31.7 (4.9)	36.3 (4.3)	35.4 (6.9)
UM-Mean (SD)b)	22.0 (8.1)	31.5 (5.6)	29.7 (5.3)	34.0 (5.1)	35.4 (6.2)

Note: N = 334;

** p<.01;

*** p<.001;

a) Mean and standard deviation of each one of the personality dimensions by empathy score top (N = 169) and bottom group (N = 165);

b) Mean and standard deviation of each one of the personality dimensions by university, UBI: N = 115; UAlg: N = 51 and UM: N = 168.

### Binary Logistic Regression

Table 3 presents the predicted coefficients (B), the coefficients standard errors (S.E), the Wald statistics (χ^2^ Wald), the significance level (p), the odds ratios [Exp (B)], and the 95% confidence intervals (CI) for each predictor of the logit regression model.

**Table 3:** Logit Regression results for predicting medical students' self-reported empathy.

Logit Regression	B	S.E.	χ^2^_wald_ (1)	pa)	Exp(B)	CI _95%_ Exp(B)
**Panel A**						
UBI	−.625	.254	6.063	.014	.535	[.325;.880]
UAlg	.660	.444	2.210	.137	1.935	[.811; 4.619]
Gender	−.781	.241	10.493	.001	.458	[.285; .735]
Age	−.003	.031	.011	.917	.997	[.939; 1.059]
Pseudo-R^2^_(Nagelkerke)_	.064					
χ^2^_(4)_	22.25***					
AIC	445.69					
BIC	468.47					
**Panel B**						
UBI	−.680	.275	6.118	.013	.507	[.296;.868]
UAlg	.736	.476	2.391	.122	2.087	[.821;5.301]
Gender	−.494	.287	2.959	.085	.610	[.348;1.071]
Age	−.041	.033	1.549	.213	.959	[.899;1.024]
Neuroticism	.015	.020	.549	.459	1.015	[.976;1.055]
Extraversion	.028	.024	1.317	.251	1.028	[.980;1.078]
Openess	.073	.024	8.984	.003	1.076	[1.026;1.129]
Agreablenes	.089	.029	9.794	.002	1.094	[1.034;1.157]
Conscientiousness	.026	.023	1.258	.262	1.026	[.981;1.074]
Pseudo-R^2^_(Nagelkerke)_	.168					
χ^2^_(9)_	59.59***					
AIC	417.66					
BIC	459.42					

a) p = p-value; N = 329;

*** p<.001.

The predictive power of the two panels revealed an improvement from the Nagelkerke pseudo R^2^ of 6.4% in the Panel A to 16.8% in the Panel B. Through the differences in the chi-square statistic and in the degrees of freedom of the two panels, we found the predictive power improvement as statistically significant (p<.001), according to the chi-square table: Δ χ^2^ = 59.59−22.25 = 37.34; Δ df = 9−4 = 5. The Nagelkerke pseudo R^2^ of 16.8% in the Panel B indicated a model that accounted for 16.8% of the total variance, suggesting the set of predictors discriminated between students in the bottom and top empathy scores sub-samples.

Regarding to associations between personality and empathy, Wald test showed that personality dimensions Openness to Experience (OR = 1.076, χ^2^Wald (1) = 8.98, p = .003) and Agreeableness (OR = 1.094, χ^2^Wald (1) = 9.79, p = .002) were statistically significant predictors of empathy JSPE-spv scores after controlling for university, gender and age. For each five point increase in the Openness to Experience score, there was a 1.44 times greater chance of being in the top empathy score tercile when university, age and gender were controlled. Similar results for Agreeableness were obtained: for each five points increase there is a 1.56 times greater likelihood of having high empathy scores, controlling the other variables in the model.

UBI variable showed a negative impact on the probability of student being classified as top empathy score (OR = 0.507, χ^2^Wald (1) = 6.118, p = .013): being a UBI student, versus UM student, decreased by 49.3% the odds of having high empathy scores. Furthermore, the odds of having high empathy scores were four times higher in UAlg students when compared to the UBI students (OR = 1.415; χ^2^Wald (1) = 7.82, p = .005).

The logistic regression model classification power revealed an overall hit rate of 68.7% (a 19% increase compared to the proportional percentage of correct classification by chance: [(161/329)^2^+(168/329)^2^]×100 = 50%), which represented an improvement over chance index of 37.4% ([(68.7%−50%)/(1–50%)] * 100). According to this result, the model provided a 37.4% reduction in overall classification error over chance, which means 37.4% less classification errors than those made if classification was done by chance. Correct prediction rates of 70.2% for the most empathic students (Sensitivity) and 67.1% for the least empathic students (Specificity) were found. This improvement was significant at p<.001, according to a one proportion test.

**Figure 1:**
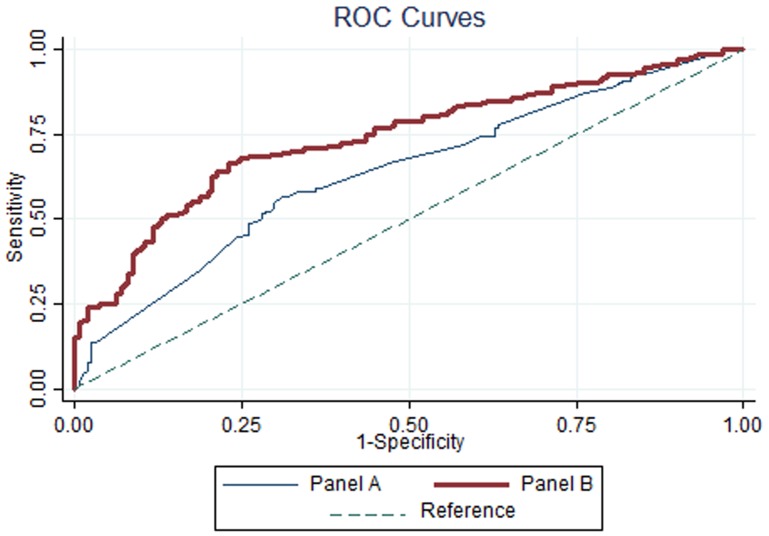
ROC curves predictive logit model for empathy (Panel A and Panel B).

Concerning to the ROC, Panel B model presented an area under the curve (AUC) of .74, which was significantly higher than 0.5 (p<.001) and significantly different (p<.001) from the .64 AUC of Panel A model (Figure 1). This suggested that the two models were significantly different in their predictive ability and that Panel B presented a reasonable predictive ability to classify students in the Bottom or Top empathy score group.

**Figure 2:**
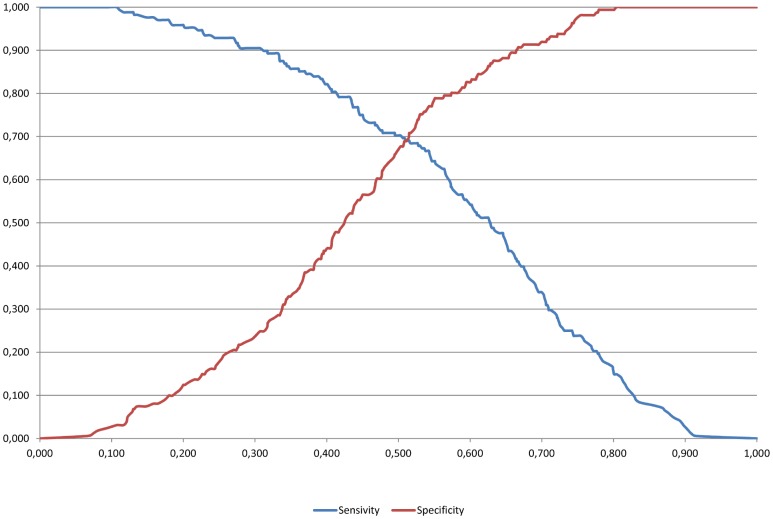
Optimal cut-off value using the sensitivity and specificity of the Panel B logit model.

If the optimal cut-off value of .508 was considered (Figure 2), then the model would accurately classify 69.6% of students in Top (Sensitivity) and 68.9% of students in Bottom group (Specificity). The hit rate would increase to 69.3%, which according to a binomial proportion test was significantly higher than 50% (p<.001).

## Discussion

The present multi-institutional and cross-sectional study in Portugal suggested that medical students who were more agreeable and open to experience were also likely more empathetic. This conclusion reinforces the argument that, personality and empathy of medical students are related [16], [18], [32]–[34] and confirms the specific findings for Portugal of a former study conducted in one of the institutions [18]. Participants were both high school entry and graduate entry students, from a range of 3 geographically distant schools with different program structures. There are no published multi-institutional studies that contemplate such diversity of participants.

Our findings showed that personality made a significant contribution to identify the more empathic students since inclusion of the Big5 Personality dimensions in our model resulted in gains in the predictive power of approximately 10%. The key contributing personality dimensions were Agreeableness and Openness to Experience, which are considered to be favorable for medical students, particularly in the clinical environment [35]–[38] as facilitators for establishing good rapport in the doctor/patient relationship and in dealing with the unexpected. The absence of a significant association between empathy and conscientiousness, contrary to what we expected, suggests that the two constructs are independent, even though conscientiousness may be the key to performance in the working environment [39]–[42].

The contribution of gender differences to assign individuals to the lowest/highest tercile groups of empathy scores was poor and not statistically significant. However, tests of associations between gender and age with empathy revealed significant gender differences - females outscored males – as reported in the majority of empathy studies [43] and age made no significant differentiation. This lead us to conclude that further important variables beyond gender, age and university are needed to explain the empathy levels of medical students.

Additionally, inter-institutional comparisons revealed that the JSPE-spv scores of medical students differed between medical schools, with the highest and lowest scores (significantly different) corresponding to, respectively, UAlg and UBI. UM and UBI scores also differed significantly but UM and UALg were not. It was interesting to notice that 32.1% of the UM and UAlg participants were graduate entry students, who had gone through admissions process in the corresponding institutions with common elements: the Multiple Mini Interview (MMI). The UBI does not apply the MMI. Taken together, since the pool of graduate entry candidates is potentially the same for all schools as the process is open to all Portuguese citizens, these findings suggest that there was a positive contribute of MMIs to attract or to select students with enhanced empathy. Indeed it has been reported that students with high levels of Conscientiousness and Agreeableness are being attracted to schools that use interviews in their selection process [24]. That evidence combined with our findings that the most agreeable and conscientious students are also the most empathic, justify our result that schools that use MMIs have the most empathic students. An implication of this study is that feasible selection methods based on interviews may discriminate positively students who will be more empathetic.

Our study is necessarily sensible to limitations, the major being the use of self-reported measures like empathy and personality, which are necessarily different from measurements from observations of the student when communicating with patients. Another limitation is related to the low predictive power of the regression analysis presented. More than 80% of empathy scores' total variance remained unexplained, which means there is a set of empathy predictors that was not yet discovered. Nevertheless, the model classified students into the Top and Buttom empathy score groups with 37.4% less classification errors than those made if classification was done by chance.

We are also aware that our sample is not representative of the Portuguese population and medical students across a long time span. However, we provide unique multi-institutional data from one country with a Latin culture that we feel as important to advance our understanding on the associations between empathy and personality of medical students.

Naturally gender and age are variables that are outside the range of the educational interventions, but there may be aspects for personality that are amenable to change. Interesting, other variables need to be explored to predict the empathy of medical students with greater accurateness. Those are probably the ones which are teachable [13] and may make students respond to interventions such as video clip discussions [12] [44], writing interventions [45], communication skills training [44] or engaging students in the creative arts [44].
